# An Audit of Core Trainees' Preferences of Teaching Format for Weekly MRCPsych Teaching at HPFT

**DOI:** 10.1192/bjo.2024.285

**Published:** 2024-08-01

**Authors:** Ariela Carno, Afifa Alihassan

**Affiliations:** Hertfordshire Partnership University NHS Foundation Trust, Hertfordshire, United Kingdom

## Abstract

**Aims:**

The primary aim was to establish the preferences of the majority of core trainees regarding online, in-person, or hybrid teaching in order to assess if the online format created during the COVID 19 Pandemic should be maintained.

Secondary aims were:

- To collect feedback regarding the barriers to in-person teaching.
- To collect feedback regarding the course content.
- To alter the way the course is presented (if required) and to incorporate the feedback regarding the course content into the course.
- To re-audit to see if the intervention was successful.

**Methods:**

- Surveymonkey was used to generate an online survey with 5 questions.
- There were a mix of quantitative and qualitative questions.
- Responses were collected between 26^th^ September 2022 and 10^th^ October 2022 and results were presented at the Tutors Committee Meeting and Junior Doctors Forum.
- Changes were implemented in the curriculum:
  1. Introduced specific neuroscience teaching.
  2. Small exam specific study groups were encouraged.
  3. It was decided that teaching would remain hybrid as per the majority preference and to allow equal access to teaching for all trainees (as per the GMC guidance).

A second survey with the same questions was sent out and responses collected between 19^th^ November 2023 to 29^th^ November 2023 to establish whether opinions had changed and to see if the intervention was successful.

**Results:**

Sept – Oct 2022

- There were 20 responses overall.
- 50% (n = 10) preferred online teaching; 45% (n = 9) preferred hybrid; 5% (n = 1) preferred in-person.
- The most common barriers to in-person teaching were the difficulty in finding parking (70%, n = 14), and being unable to leave work on time due to clinical responsibilities (50%, n = 10).
- The most common preferred frequency of in-person attendance for the hybrid model was monthly (45%, n = 9).
- Topics requested to be covered (free-form question) included psychopharmacology, CAMHS, perinatal, geriatric, neuroanatomy and neuroscience.

Nov 2023

- There were 22 responses overall, including new trainees that had not done the survey last year.
- 50% (n = 11) preferred online teaching, 41% (n = 9) preferred in-person; 9% (n = 2) preferred in-person.
- The most common barriers were the same: difficulty finding parking (64%, n = 14) and clinical responsibilities (55%, n = 12).
- It was commented that neuroscience related teaching had improved.

**Conclusion:**

There was a clear preference in both surveys amongst trainees for either online or hybrid teaching formats. Hence a decision was made to continue the current format of flexible teaching.

